# The role of recurrent somatic mutations that alter conserved m^6^A motifs in human cancer

**DOI:** 10.1093/narcan/zcaf014

**Published:** 2025-04-23

**Authors:** Oliver Artz, James R White, Benoit Rousseau, Guillem Argiles, Michael B Foote, Paul Johannet, Miteshkumar Patel, Somer Abdelfattah, Shrey Patel, Callahan Wilde, David Mieles, Luis A Diaz

**Affiliations:** Di vision of Solid Tumor Oncology, Department of Medicine, Memorial Sloan Kettering, New York City, NY 10065, United States; Resphera Biosciences, Baltimore, MD 21231, United States; Di vision of Solid Tumor Oncology, Department of Medicine, Memorial Sloan Kettering, New York City, NY 10065, United States; Di vision of Solid Tumor Oncology, Department of Medicine, Memorial Sloan Kettering, New York City, NY 10065, United States; Di vision of Solid Tumor Oncology, Department of Medicine, Memorial Sloan Kettering, New York City, NY 10065, United States; Di vision of Solid Tumor Oncology, Department of Medicine, Memorial Sloan Kettering, New York City, NY 10065, United States; Di vision of Solid Tumor Oncology, Department of Medicine, Memorial Sloan Kettering, New York City, NY 10065, United States; Di vision of Solid Tumor Oncology, Department of Medicine, Memorial Sloan Kettering, New York City, NY 10065, United States; Di vision of Solid Tumor Oncology, Department of Medicine, Memorial Sloan Kettering, New York City, NY 10065, United States; Di vision of Solid Tumor Oncology, Department of Medicine, Memorial Sloan Kettering, New York City, NY 10065, United States; Di vision of Solid Tumor Oncology, Department of Medicine, Memorial Sloan Kettering, New York City, NY 10065, United States; Di vision of Solid Tumor Oncology, Department of Medicine, Memorial Sloan Kettering, New York City, NY 10065, United States

## Abstract

N^6^-methyladenosine (m^6^A) is the most abundant internal RNA modification in eukaryotes and plays a key role in cellular growth and development. Global changes in cellular methylated RNA and m^6^A-mediated transcript regulation significantly impact oncogenesis. Here, we investigate how recurrent synonymous and non-synonymous somatic mutations abolishing individual canonical methylated m^6^A motifs affect transcript levels and survival of patients with cancer. Moreover, we explore the effect of these mutations on creating *de novo* m^6^A motifs. To this end, we compared publicly available data on m^6^A sites with mutations reported in The Cancer Genome Atlas (TCGA). We find that mutations disrupting or creating m^6^A motifs display a low recurrence and have a negligible impact on RNA abundance. Patients with the highest number of disrupted m^6^A sites or newly generated m^6^A motifs did not generally exhibit alterations in mortality risk or outcomes. Hence, our data suggest that mutational alterations in the m^6^A motif landscape are unlikely to be a primary mechanism for regulating gene function across most cancer types. This may be attributed to the fact that mutations typically affect individual m^6^A sites, which is likely insufficient to significantly impact gene expression.

## Graphical Abstract

**Graphical Abstract:**
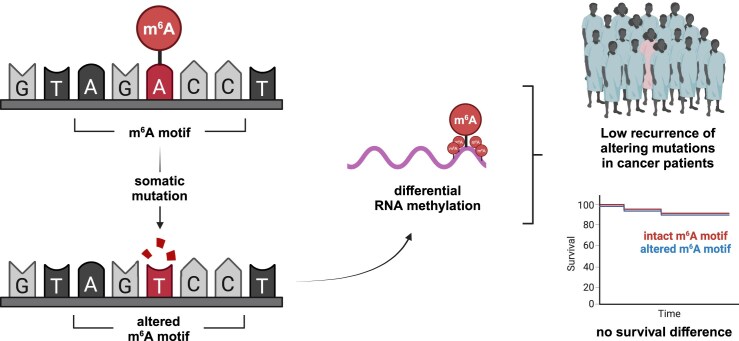

## Introduction

Chemical modification of biological macromolecules is an essential feature of gene regulation. More than 100 different modifications have been described for RNA of which N^6^-methyladenosine (m^6^A) is the most abundant internal mRNA modification in eukaryotes [1]. M^6^A plays a role in various physiological processes such as transcript stabilization, splicing, and translation, with the most significant effects being observed in mediating transcript destabilization [2, 3]. The deposition of m^6^A on mRNA is carried out by writer proteins with methyltransferase activity. The steady-state levels of m^6^A are then regulated through the interplay of these writers and eraser proteins, which possess demethylase activity. The majority of m^6^A modifications occur in the context of a DRACH (D = A/U/G, R = A/G, H = A/U/C) motif [4]. Importantly, not every DRACH motif is methylated *in vivo* indicating tight regulatory mechanisms for m^6^A deposition. Moreover, m^6^A distribution along transcripts is biased towards higher densities in the last exon, 3′UTR regions, and long introns [5], likely driven by the exon junction complex [6]. Modified transcripts are recognized by reader proteins interacting with m^6^A and eliciting downstream effects through protein–protein interactions with different effectors [7]. Misregulated or mutationally disrupted writers, erasers, and readers have been associated with various cancer types affecting oncogenes, tumor suppressors, and the tumor microenvironment directly and indirectly [8]. Altering the function of machinery components generally leads to global changes in the cellular m^6^A landscape impacting a plethora of transcripts simultaneously and thus eliciting pleiotropic effects. However, our understanding of point mutations influencing m^6^A deposition on a specific transcript is limited: The mutational gain of an m^6^A site can have significant physiological effects. A G > C mutation in codon 273 of TP53 (tumor protein 53), one of the major tumor suppressor genes, creates an m^6^A site. This leads to increased p53 R273H translation, which is a putative mechanism for acquired multidrug resistance in colon cancer [9]. Furthermore, mutation of the m^6^A site A783 in the long non-coding RNA (lncRNA) HOX transcript antisense RNA (HOTAIR) results in the loss of cancer-promoting effects in cell lines [10]. Importantly, it is unknown whether these mutational events frequently occur in cancer patient cohorts to drive malignancy.

This study focuses on the impact of somatic point mutations that disrupt or create specific m^6^A sites without leading to a perturbation of the machinery itself. We investigated how mutations in DNA potentially disrupt m^6^A RNA motifs to affect cancer growth and development. To this end, we combined mutational data from The Cancer Genome Atlas (TCGA) with experimentally validated m^6^A sites from human tissue or cell lines at a single-nucleotide resolution [11–16]. Combining m^6^A sites from data sets that were generated using the most recent transcriptome-wide methods of m^6^A detection such as DART-seq [16] and GLORI-seq [13] enables comprehensive mapping of m^6^A sites in human tissues. Moreover, we investigated the potential effect of mutations that lead to a *de novo* generation of m^6^A motifs across the TCGA cohort and correlated recurrently gained motifs with transcript abundance and patient survival. Importantly, while most studies investigating the impact of mutations on protein function focus on non-synonymous mutations, this analysis includes synonymous mutations and mutations in 3′UTR regions, which have the potential to meaningfully alter the m^6^A methylome.

## Materials and methods

### Data sources and preprocessing

All m^6^A sites were derived from high-throughput sequencing experiments providing single base-pair resolution of methylated adenosines. A detailed description of all data sets can be found in Supplementary Table S1. When appropriate, genomic loci were lifted over from the genome build hg19 to GRCh38 using the R package rtracklayer (v1.54.0). Gene annotation was performed using the R package ChIPseeker (v1.30.3). Cancer gene annotations were derived from OncoKB [17]. Somatic mutations were downloaded from The Cancer Genome Atlas (TCGA) MC3 [18]. Patients with hypermutated samples considered Microsatellite Instability High (MSI-H) or harboring a Polymerase ϵ (POLE) driver mutation were removed from the data set (Supplementary Table S2).

### Motif discovery

Discovery of enriched motifs was performed using findMotifsGenome.pl from HOMER (v4.11) [19] with the following flags: -rna -len 6 -p 7.

### Differential gene expression analysis

Transcriptome count data of primary tumors was downloaded from TCGA. For each recurrently mutated gene (gene-level analysis) or motif (motif-level analysis), patients with disrupted m^6^A motifs were compared to patients with intact m^6^A motifs of the same cancer type. Differential gene expression analysis was only performed if expression data of at least two patients in the mutated group were available. DESeq2 was used for normalization and determination of differential expression across all genes in patients with mutated or intact m^6^A sites. Results were filtered by the respective gene of interest to investigate whether m^6^A site disruption significantly affects transcript abundance.

### Survival analysis

Survival data were downloaded from TCGA using the R package RTCGA (v1.24.0). Patients with high m^6^A mutational load (above the third quartile) were compared to patients with low m^6^A mutational load (below the first quartile). Only patients with at least five total mutations were considered in this analysis. Kaplan–Meier estimates and corresponding *P*-values were determined using the R packages survival (v3.5-5) and survminer (v0.4.9).

### Statistical analysis and plotting

Data were processed and analyzed using R (v4.1.3) and the following packages: BiocParallel (v1.28.3), biomaRt (v2.50.3), BSgenome (v1.62.0), cowplot (v1.1.1), data.table (v1.14.8), ggpubr (v0.6.0), ggsci (v3.0.0), pacman (v0.5.1), readxl (v1.4.2), and tidyverse (v2.0.0)

## Results

### M^6^A sites are mutated across cancer types in TCGA

**Figure 1.**
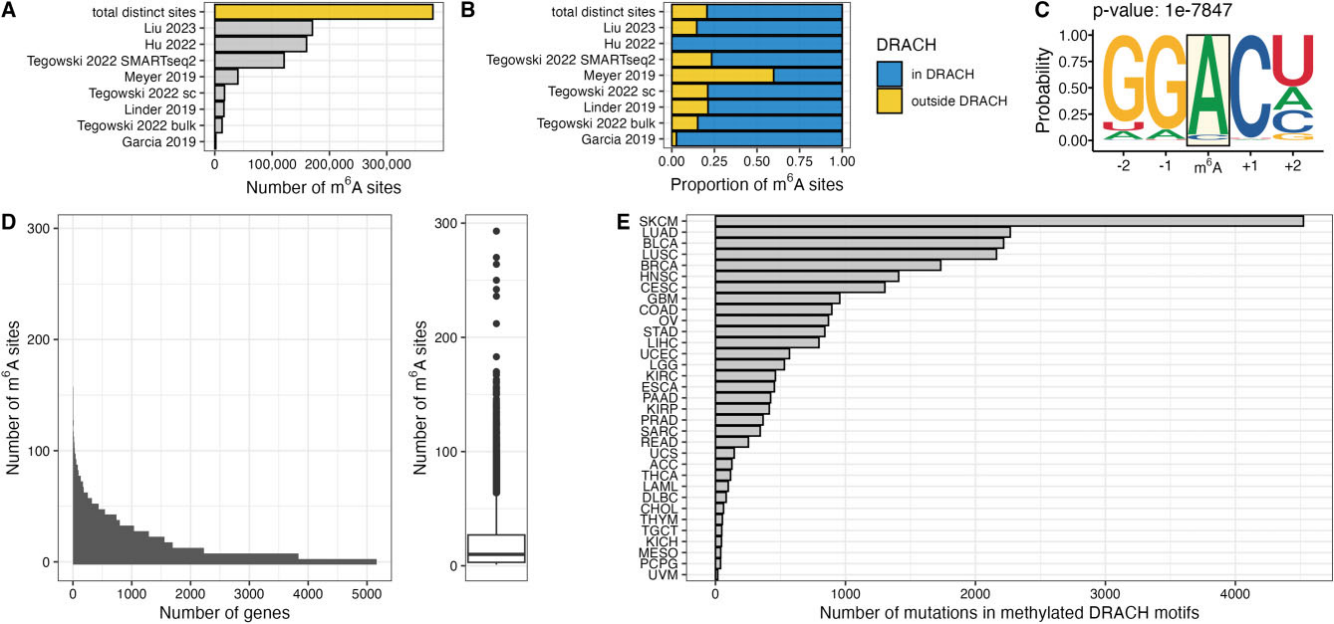
m^6^A sites and overlap with TCGA mutations. (**A**) Number of distinct m^6^A sites in each study and the total number of distinct m^6^A sites in the merged data set (top bar). (**B**) Proportion of m^6^A sites in the canonical DRACH motif. (**C**) Motif discovery in the merged data set. The probability of a given base is indicated for two bases upstream (−2, −1) and downstream (+1, +2) of the methylated adenosine (m^6^A, orange box). The *P*-value of this motif was determined by HOMER and is indicated above the sequence. (**D**) Number of identified m^6^A sites per gene. (**E**) Number of mutations from different TCGA data sets overlapping with methylated DRACH motifs.

To study the effect of mutational m^6^A site disruption, we first collected publicly available data sets identifying m^6^A sites in human samples at base-pair resolution. We combined eight individual data sets from six different publications employing various methods of transcriptome-wide m^6^A detection to construct a comprehensive map of the human m^6^A methylome (Fig. 1A and Supplementary Table S3). In total, 380 541 distinct m^6^A sites across 20 413 genes were identified. M^6^A is mostly deposited within the context of the canonical DRACH (D = A/U/G, R = A/G, H = A/U/C) motif [4]. While the enrichment of identified m^6^A sites within this motif varies between data sets (Fig. 1B), we find that in our combined data set, 79% of m^6^A sites are within this consensus motif. Motif enrichment analysis confirms that the DRACH motif is significantly enriched in this data set (Fig. 1C). Total numbers of identified m^6^A sites vary across genes ranging up to 293 sites in XIST (X-inactive specific transcript) with a median of 10 sites per gene (Fig. 1D). The distribution of the number of identified m^6^A sites is consistent in oncogenes and tumor suppressors (Supplementary Fig. S1A). Oncogenes display a statistically significant (*P*= 0.001) lower number of m^6^A sites per gene than tumor suppressor genes (22.5 versus 28 median m^6^A sites per gene). The difference remains statistically significant after normalizing for transcript length (*P*= 0.041) (Supplementary Fig. S1B).

Analysis of mutations reported in TCGA revealed that depending on the cancer type, between 17 (TCGA–UVM) and 4524 (TCGA–SKCM) (median per cancer type: 424) mutations overlap with DRACH motifs that were identified to be methylated (Fig. 1E). Importantly, some of these mutations disrupt canonical DRACH motifs, likely resulting in a loss of methylation. We included single nucleotide variants classified as missense mutations, synonymous mutations, and mutations in the 3′UTR regions of genes. Synonymous mutations and mutations in 3′UTR regions generally do not have a direct effect on the translated polypeptide sequence outside their effect on splicing or alternative polyadenylation. We hypothesized, however, that these variant types have a profound effect on gene function by altering the epitranscriptome, in particular m^6^A deposition. We identified up to 118 genes that have a disrupted m^6^A motif in at least two patients per cancer type (Supplementary Fig. S1C). Up to 19 specific motifs are mutated in at least two patients per cancer type (Supplementary Fig. S1D).

### Mutations in m^6^A sites exhibit low recurrence

**Figure 2.**
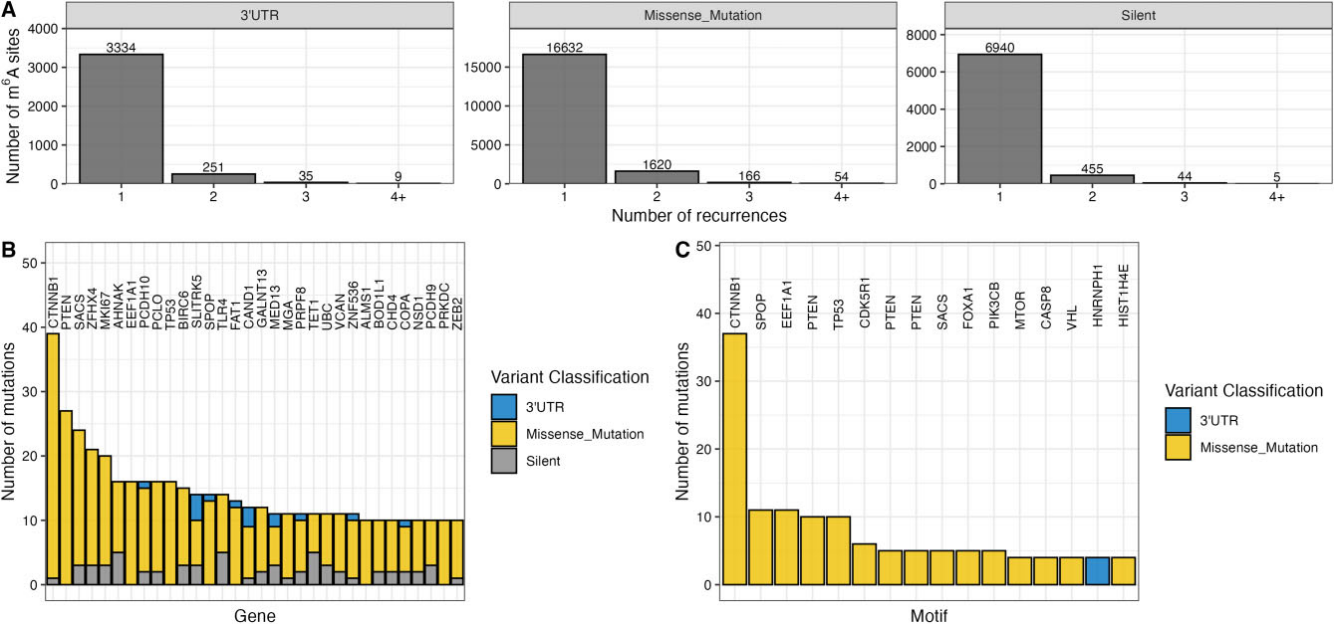
Recurrence of mutations in m^6^A sites. (**A**) Recurrence of mutations overlapping with m^6^A motifs for mutations classified as 3′UTR, missense, or silent mutations. (**B**) The number of mutations overlapping with m^6^A motifs per gene. (**C**) The number of mutations overlapping with a specific m^6^A motif. Every bar represents a specific m^6^A motif. The annotation indicates in which gene the motif is located.

A key factor for assessing the relevance of a mutation in driving disease is its recurrence in the patient population. Most mutations disrupting m^6^A motifs were observed as singular events in one patient across TCGA (Fig. 2A). We investigated the number of m^6^A motif-disrupting mutations per gene for each patient (Supplementary Fig. S2A). In our data, 98.5% of genes across patients with a mutationally disrupted m^6^A motif harbor only one disrupting mutation. The highest number of m^6^A disrupting mutations in a single gene is four and was observed in only 0.01% of genes across patients. This suggests that in an individual patient, no more than a single m^6^A motif is mutated in most genes. Several oncogenes and tumor suppressors such as CTNNB1 (catenin β1), PTEN (phosphatase and tensin homolog), or TP53 are among the genes with the highest number of disrupted m^6^A sites (Fig. 2B). Similarly, the highest recurrent disruptions of specific motifs occur in oncogenes or tumor suppressors (Fig. 2C). Interestingly, the m^6^A mutational burden in CTNNB1 is mainly driven by the disruption of one specific motif. We next compared the recurrence of mutations in DRACH motifs harboring m^6^A to DRACH motifs that are not reported to be methylated (Supplementary Fig. S2B). Cancer genes do not exhibit significant differences between the recurrence of 3′UTR, missense, or silent mutations in methylated or unmethylated DRACH sites at the base or motif level. This suggests that disruptions of m^6^A motifs are neither selected for nor against in the oncogenic process. Significantly higher recurrences of mutations in m^6^A harboring DRACH motifs compared to DRACH motifs without m^6^A, however, can be observed at the gene level for both oncogenes and tumor suppressor genes. The same trend can be observed for genes neither designated oncogene nor tumor suppressor gene suggesting that the statistically significant difference might not be biologically meaningful for cancer pathology.

### Recurring mutations in m^6^A sites do not significantly influence transcript abundance

**Figure 3.**
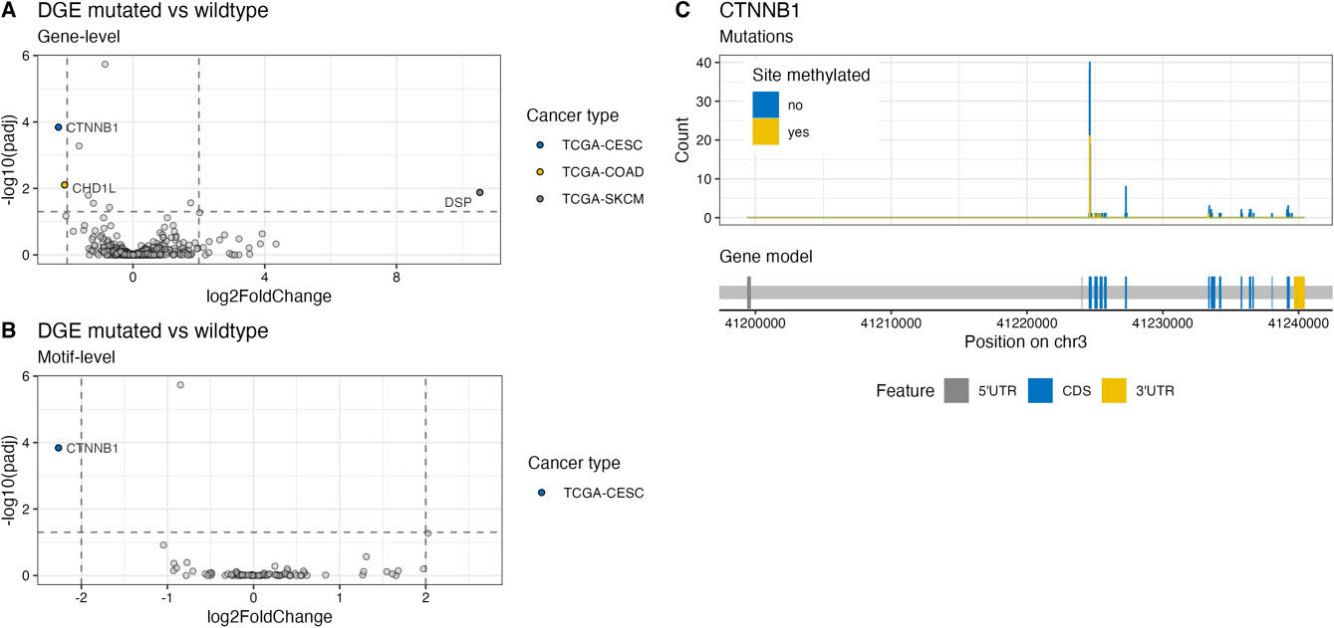
Effect of m^6^A motif disruption on gene expression. (**A** and **B**) Differential gene expression analysis comparing patients with disrupted (mutated) m^6^A motifs to patients with the same cancer type and intact (wild-type) m^6^A motifs. Significant differentially expressed genes for specific cancer types (adjusted *P*-value < 0.05 and fold change > |2|) are highlighted and annotated. (A) Gene-level analysis. Genes with disrupted m^6^A motifs in at least two patients were considered in the analysis. (B) Motif-level analysis. Specific m^6^A motifs disrupted in at least two patients were considered for the analysis. The annotated gene name corresponds to the gene harboring the motif. (**C**) Gene model of CTNNB1 (bottom) and the distribution of mutations in methylated or not methylated sites across CTNNB1 (top).

The major effect of m^6^A on mRNA is promoting degradation and thus decreasing transcript abundance [7]. While in some contexts m^6^A has been associated with transcript stabilization, in most cases the dominant effect is transcript de-stabilization. To test whether the loss of m^6^A motifs and subsequent lack of methylation influences transcript abundance, we analyzed the gene expression of patients from TCGA. We analyzed the expression of genes that harbor a mutation disrupting an m^6^A site in at least two patients of the same cancer type. The expression was compared to all other patients with intact m^6^A motifs of the same cancer type (Fig. 3A). Three genes across three different cancer types are significantly misregulated. Upon motif disruption, CHD1L (chromodomain helicase DNA binding protein 1-like) is significantly downregulated in the TCGA–COAD cohort, CTNNB1 is significantly downregulated in the TCGA–CESC cohort, and DSP (desmoplakin) is significantly upregulated in the TCGA–SKCM cohort. The analysis of recurrently disrupted m^6^A motifs on the motif level revealed that one motif in CTNNB1 in the TCGA-CESC cohort is associated with reduced transcript abundance (Fig. 3B). Further inspection of m^6^A motifs disrupted in CTNNB1 revealed that mutations in these loci not only interfere with m^6^A deposition but also impact CTNNB1 protein degradation: Mutations in D32 or S33 stabilize b-catenin protein by interfering with ubiquitin-dependent proteasomal degradation [20]. Our analysis indicates the presence of a mutational hotspot region with mutations in methylated or unmethylated sites (Fig. 3C). Taken together, the observed correlation between m^6^A site disruption and expression alteration does not imply causality. Mutations in this hotspot might have significant effects on protein abundance and thus protein function independent of m^6^A.

### M^6^A mutational burden does not affect patient survival

**Figure 4.**
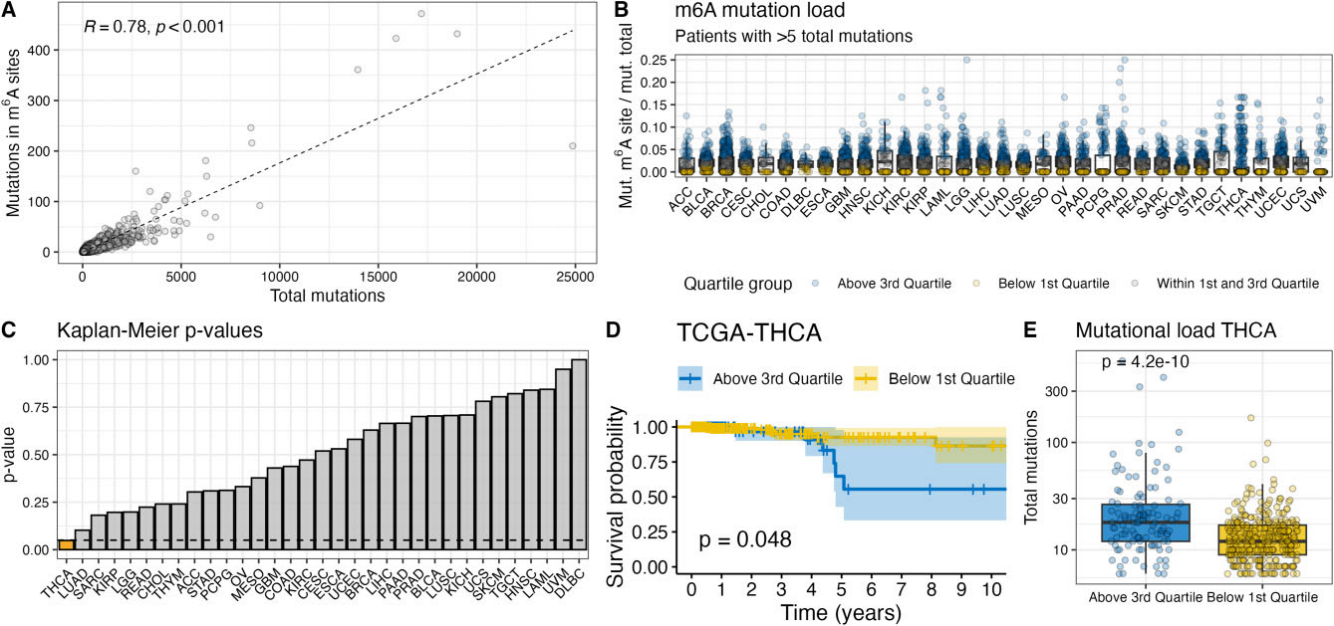
Effect of m^6^A mutational burden on patient survival. (**A**) Spearman correlation between total mutations and mutations in m^6^A sites. (**B**) M^6^A mutational burden of patients for different cancer types across TCGA. Only patients with more than five total mutations were considered in the analysis. (**C**) *P*-values of Kaplan–Meier estimates comparing survival of patients with high and low m^6^A mutational burden. (**D**) Kaplan–Meier curve for patients with high (above third quartile) and low (below first quartile) m^6^A mutational burden in TCGA–THCA (thyroid carcinoma). (**E**) Comparison of total mutational burden in TCGA–THCA. The *P*-value was determined using the Mann–Whitney *U* test.

To date, it is unclear whether a single m^6^A site is sufficient to exert a physiologically relevant molecular function. While the disruption of a single site can influence transcript splicing [9, 21], the clustered occurrence of m^6^A sites as recently described by Liu *et al.* [13] suggests a redundancy of specific sites within a cluster. Consequently, the disruption of a single site within a cluster might not have a strong physiological effect because m^6^A reader proteins would still be able to bind the same transcript at a different m^6^A site within the cluster. We determined the m^6^A mutation load by normalizing all mutations in m^6^A sites by the number of total mutations per patient to assess the effects in patients with many disruptions. Since we include mutations in 3′UTRs and synonymous mutations in m^6^A sites, these variant classes were also included in the calculation of total mutations. The number of mutations in m^6^A sites strongly correlates with the number of total mutations for most patients (*R*^2^= 0.78, *P*< 0.001) (Fig. 4A). We separated patients into groups with high or low m^6^A mutational load for each cancer type and analyzed their overall survival rates (Fig. 4B). A significantly different overall survival rate was found for papillary thyroid carcinoma (TCGA–THCA, *P*= 0.048), but not any of the other investigated cohorts (Fig. 4C). Patients with papillary thyroid carcinoma and a low m^6^A mutation burden exhibited higher survival rates than those with a high m^6^A mutation burden (Fig. 4D). However, patients with a high m^6^A burden also had a significantly higher total mutation burden (*P*= 4.3 × 10^−10^) (Fig. 4E). The survival benefit might therefore not be attributed to the observed effects on the epitranscriptome, but to other well-established predictors of clinical response such as tumor mutational burden (TMB) [22] or tumor stage.

### Mutational gain of m^6^A motifs does not significantly affect gene expression or patient survival

**Figure 5.**
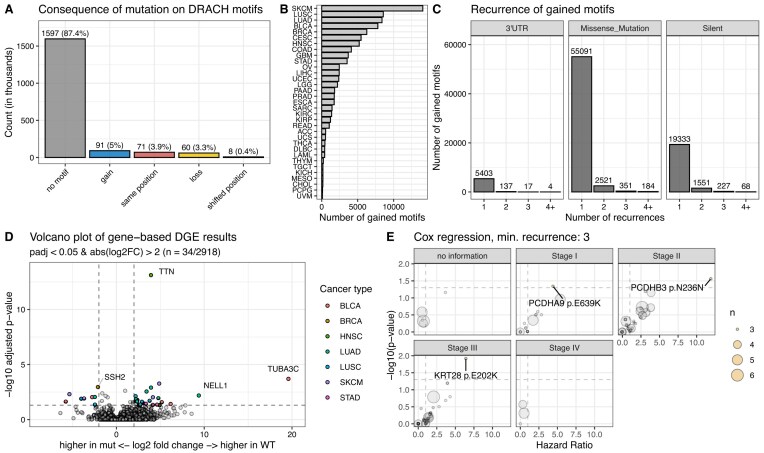
Description and effect of gained DRACH motifs. (**A**) Consequences of mutations on DRACH motifs across all examined mutations. (**B**) Number of gained DRACH motifs per cancer TCGA cancer type. (**C**) Recurrence of 3′UTR, missense, or silent mutations leading to DRACH gains. (**D**) Gene-level analysis of transcript abundance. Genes with gained m^6^A motifs in at least two patients were considered in the analysis. (**E**) Survival analysis of mutations leading to DRACH gain stratified by cancer type and stage. Only mutations occurring at least three times per cancer type and stage were considered in the analysis.

Our analyses above focused on the effect of mutations in DRACH motifs that were experimentally validated to be methylated in at least one of the surveyed data sets. To understand whether the mutational gain of m^6^A sites could potentially affect cancer growth and development, we investigated all missense, silent, and 3′UTR mutations across the TCGA cohort. There are five potential consequences of mutations regarding DRACH motifs (Fig. 5A): Most mutations were not within DRACH motifs (no motif’, 87.4%). ‘Gain’ describes a scenario in which a mutation generates a DRACH motif *de novo* in a position that was previously no DRACH motif. We observed this effect in 5% of mutations. In approximately 4% of cases, an existent DRACH motif was altered but retained after mutation due to the redundancy in certain positions of the motif (“same position”). DRACH motifs were disrupted in 3.3% of cases (“loss”) and a small fraction of mutations (0.4%) led to a retained but shifted DRACH motif (“shifted position”). For further analyses, we only considered *de novo* generated (“gain”) motifs.

Gained motifs were identified across 33 cancer types with the highest number in SKCM (*n* = 14 058) and the lowest number in UVM (*n* = 99) (median: 1591) (Fig. 5B). Similar to our observations in lost m^6^A sites, the recurrence of gained DRACH motifs is relatively low across mutation types (Fig. 5C). The majority of gained motifs were observed once in the TCGA cohort. Few gained motifs were observed four times or more (4 motifs mediated by 3′UTR mutations, 184 motifs mediated by missense mutations, and 68 motifs mediated by silent mutations). We performed differential gene expression analysis to see if mutationally gained DRACH motifs impact transcript abundance. We analyzed the expression of genes that harbor a mutation generating an m^6^A site in at least two patients of the same cancer type. The expression was compared to all other patients of the same cancer type (Fig. 5D). Of the 2943 surveyed genes, 34 were significantly differentially expressed (adjusted *P*-value < 0.05, absolute log2 fold change > 2) (Supplementary Table S4). Since the predominant role of m^6^A is transcript destabilization, a gained motif in the mutant sequence should lead to lower transcript abundance. Down-regulation after motif gain was observed in 26 of the identified differentially expressed genes. The recurrence of mutations in these genes is between 3 and 31 (Supplementary Fig. S3A). TTN (titin) and CSMD3 (CUB and sushi multiple domains 3) exhibit the most mutations (*n* = 31 and *n* = 19), which is likely due to their large coding regions and unlikely to be selected for in cancer cells by gaining a fitness advantage. Focusing on the effect of a specific gained motif instead of the cumulative effect per gene, we found that no single gained motif had a significant effect on transcript abundance (Supplementary Fig. S3B). Analogously to lost motifs, we investigated the effect on overall survival stratified by cancer type and stage (Fig. 5E). We performed Cox regressions on patient survival data based on mutational status. We only considered motifs gained in at least three patients. We did not identify survival differences in patients with Stage IV disease. Three mutational gains showed a survival decrement (Supplementary Fig. S3C). Only three patients were identified harboring each mutation indicating a low recurrence in the patient population. Lastly, we investigated the mutational load of gained DRACH sites per patient (Supplementary Fig. S3D). Most transcripts in patients do not harbor more than a single gained DRACH motif (>99%). Across the entire population, there were only 871 instances in which a patient’s gene had more than one gained DRACH motif.

## Discussion

M^6^A plays an important role in cancer growth and development [8]. To date, most studies investigate the effect of m^6^A machinery perturbations, which affect many transcripts simultaneously leading to pleiotropic effects. Here, we investigated the effect of somatic mutations disrupting individual m^6^A sites across the entire transcriptome for different cancer types. Moreover, we survey the effect of possible newly gained m^6^A sites. Our knowledge of mutations that do not affect the entire m^6^A landscape, but specifically disrupt or create distinct sites, is limited. Notably, the mutational disruption of m^6^A sites leads to a qualitative change in methylation status irrespective of the native stoichiometry. Therefore, relatively strong effects are predicted. Moreover, mutationally gained DRACH sites may lead to new m^6^A sites and potential transcript destabilization, as previously observed [9]. We hypothesized cancer development might be driven by somatic mutations that alter m^6^A sites, thus affecting gene function.

To test the effect of motif disruption, we combined mutational data from TCGA with publicly available data sets identifying m^6^A sites at single-base resolution. In addition to missense mutations, we included synonymous mutations and mutations in 3′UTR regions of transcripts in our analysis. Since neither of these two types directly impacts the amino acid sequence of a protein, they are often disregarded in mutational analyses despite having the potential to meaningfully alter RNA sequences and thus gene function.

We found that many canonical m^6^A sites across the transcriptome are disrupted by somatic mutations in different cancer types. However, these mutations do not generally occur at high frequencies in the investigated cohorts and are therefore unlikely to be primary drivers of malignancy. The most well-described molecular effect of m^6^A is transcript destabilization [7]. We tested whether mutational disruption of m^6^A sites leads to changes in transcript abundance across TCGA cohorts. Our data suggest that the loss of individual methylated DRACH sites does not generally lead to significant changes in transcript abundance in the investigated patients. One notable exception was the disruption of methylated DRACH sites in CTNNB1 being associated with a significant down-regulation of CTNNB1 transcript in the stomach adenocarcinoma cohort (TCGA–CESC). However, mutations in these codons not only influence RNA methylation but also protein abundance [20]. The observed molecular effects are therefore unlikely mediated by altered transcript methylation but by effects on the CTNNB1 protein level.

In our data set, the number of mutations in m^6^A sites strongly correlates with the total mutational burden of patients. This indicates that mutations in m^6^A sites might not be under selection to drive malignancy. When normalizing for total mutational burden, m^6^A mutational load is not prognostic for overall survival benefit in most tumor types. A notable exception are patients with a high m^6^A mutational burden in the thyroid carcinoma cohort (TCGA–THCA), who exhibit a significant overall survival benefit. However, these patients had tumors at various stages and show a significantly higher total mutational burden, which is the likely explanation for the observed survival benefit. In summary, our data provide evidence that the somatic mutational disruption of individual m^6^A sites might not play a major role in cancer growth and development.

Notably, m^6^A modifications have been described to occur in clusters akin to CpG islands in the context of DNA methylation [13]. It is feasible that m^6^A clusters harbor redundant m^6^A sites, thus evading mutational disruption and ensuring robust gene regulation. Indeed, data from *in vitro* experiments suggest that the half-life of hypermethylated transcripts correlates with the number of m6A sites per transcript [23]. Most transcripts in our data set exhibit only a single mutated m^6^A site and we find a maximum of four sites mutated per transcript. Hence, we do not identify clusters of multiple m^6^A sites in close spatial proximity to be meaningfully mutated, which might explain why somatic mutations affecting individual m^6^A sites have negligible physiological consequences. Indeed, our knowledge of individual m^6^A sites affecting gene function *in vivo* is limited, possibly due to the attenuation of mutational consequences through redundant m^6^A sites within clusters.

Gaining DRACH motifs can potentially create novel m^6^A sites leading to transcript destabilization. TCGA data allow us to identify mutationally gained DRACH motifs and investigate associations with transcript abundance and patient survival. A novel DRACH motif can be a potential target for methylation. However, it is unknown whether this site will be methylated *in vivo*. After analysis of missense mutations, synonymous mutations, and mutations in 3′UTR regions, our data suggest that the mutational gain of m^6^A sites is unlikely to be oncogenic. We found that somatic mutations frequently generate new DRACH motifs in the transcriptome across cancer types in the TCGA cohort. However, the recurrence of specific gains is low indicating stochastic processes and likely no selection for or against those gains. We identified 26 transcripts for which a DRACH gain leads to decreased abundance. Future studies will be necessary to elucidate whether this effect is indeed due to increased methylation. We also identified three gained motifs that lead to worse patient survival outcomes. Most genes harbor no or a single gained motif in each patient. As discussed for disrupted motifs, it is feasible that to affect transcript abundance, gained motifs must occur in spatial proximity forming a cluster.

There are several limitations to this study. Our analysis excludes m^6^A deposited outside canonical DRACH motifs. Hence, we are not able to discern whether mutations in those sites might be affected differently leading to more consequential molecular effects. Non-DRACH m6A sites have been reported in all m^6^A data sets that were included in this study, except for Hu*et al.* [12]. While some non-DRACH m^6^A sites might be false positives and artifacts of the employed detection method, the existence of these sites has been confirmed *in vivo* [24]. The functional relevance of non-DRACH sites, however, has not been elucidated. Our analysis encompasses the majority of m^6^A sites, with non-DRACH sites representing only 21% of the data set. Another important caveat of our approach is that m^6^A sites were not experimentally determined in TCGA samples directly. Instead, we compare TCGA data to a landscape of experimentally confirmed m^6^A sites from human samples and cell lines. While m^6^A has long been considered reversible and highly dynamic [25], more recent evidence suggests that it is “hard-coded” and determined by the gene structure [6, 11]. Therefore, m^6^A sites identified in most human samples can be compared to TCGA data. Disparities between m^6^A site data sets are more likely due to technical differences than biological dynamics. Importantly, our approach is restricted to qualitative differences in m^6^A deposition and does not account for changes in stoichiometry. This limitation, however, accurately represents the biology of somatic mutations disrupting m^6^A sites: Once disrupted, a given site can no longer be methylated irrespective of cell state or environmental context, thus a stoichiometric fine-tuning of the epitranscriptome in that site is no longer possible. This approach has the potential to generate false positive findings in low-stoichiometry sites that might not be relevant. Our findings, however, suggest that disruptions of m^6^A sites regardless of stoichiometry do not have a significant effect on cancer growth and development. Finally, our analysis focused on two major outcomes: transcript abundance and survival benefit. Although m^6^A site disruption and DRACH motif gain did not affect either of these, it is feasible that other parameters such as pre-mRNA splicing or disease progression are influenced by these factors.

While this manuscript was under review, Lan*et al.* [26] published a study providing evidence that the mutational disruption of m^6^A sites does indeed influence RNA metabolism and promote tumorigenesis. Similar to the approach described here, the authors directly compared how a large data set of m^6^A sites derived from various tissue sources might be disrupted by recurrently occurring mutations. The authors identify multiple mutations disrupting m^6^A sites that recur at higher frequencies than expected. The functional evidence presented for the two highest-ranking mutations, one in BRCA2 (breast cancer susceptibility protein 2) and one in CDKN2A (cyclin dependent kinase inhibitor 2A), strongly suggests that the mutational disruption of a single m^6^A site influences mRNA stability. The authors demonstrate that a subset of synonymous mutations can change gene function not by altering the protein sequence directly, but affecting epitranscriptomic processes that lead to differences in mRNA abundance. While investigating the same hypothesis in this study, we chose to prioritize patient outcomes to assess the role of m^6^A disruptions on tumorigenesis. We arrive at a different conclusion than Lan*et al.* because we wanted to know whether this process is a common pathway in cancer and ultimately affects patient outcomes. Upon closer inspection of the top ranked mutation (BRCA2-c.1365A > G), we noticed that Lan*et al.* report a frequency of 1.68% in prostate samples in their data set. Notably, we were not able to identify this mutation in any of the patients in our TCGA-based data set. We next checked the occurrence of this mutation in an unpublished internal MSKCC IMPACT sequencing cohort (85 343 samples) and were not able to identify the mutation either. COSMIC reports 42 prostate samples in which BRCA2-c.1365A > G occurs, all derived from one study [27]. Notably, patients in this study were highly selected and had a significant enrichment in BRCA2 mutations. Lastly, this locus has been deposited in dbSNP (rs1801439). The ALFA allele frequency in the global population is 3.5% suggesting that this mutation might be a common germline SNP (single nucleotide polymorphism) and unlikely to be strongly associated with tumorigenesis. These data are aligned with our conclusion that m6A motif disruption is not a major mechanism for tumorigenesis. The strong functional data provided by Lan*et al.*, however, suggest that in rare cases m^6^A motif disruption may be important. Future work will be necessary to ascertain if, and at what frequency, this mechanism is driving cancer development in patients.

In summary, our study provides evidence that somatic mutations disrupting or creating DRACH motifs might not be a common mechanism for cancer growth and development. Global shifts in the m6A epitranscriptomic landscape through modulation of the machinery or mutational disruptions affecting multiple sites might be necessary to elicit a meaningful molecular effect and alter gene function. Further studies are necessary to elucidate whether m^6^A clusters are a protective mechanism ensuring appropriate gene expression. Recent advances in mapping mRNA modifications at base-pair resolution on a transcriptome-wide scale, as recently demonstrated for pseudouridine [28], will pave the way for the investigation of somatic mutational disruption of other modifications. It will be interesting to see whether epitranscriptomic driver mutations can be identified that do not directly rely on modifying protein sequences. Moreover, our data set can be used as an updated resource for the community. Some previous studies combine m^6^A site data across organisms, but lack information about mutational disruption [29]. Other studies containing information about mutational disruption were published before the advent of important techniques such as GLORI-seq and therefore do not include important high-confidence m^6^A sites [30, 31].

## Supplementary Material

zcaf014_Supplemental_Files

## Data Availability

The m^6^A site data sets were derived from sources in the public domain. Please see Supplementary Table S1 for details. Mutational data are available from the TCGA Research Network: https://www.cancer.gov/tcga. Code used for analyses has been uploaded to Zenodo (DOI: 10.5281/zenodo.15101536).
