# Supplementary material for: The role of recurrent somatic mutations that alter conserved m6A motifs in human cancer
Source: NAR Cancer. 2025 Apr 23;7(2):zcaf014. doi: 10.1093/narcan/zcaf014 (PMC12015683; doi:10.1093/narcan/zcaf014)
Supplement: zcaf014_Supplemental_Files [file zcaf014_supplemental_files.zip › Artz_m6A_supp.pdf]

The role of recurrent somatic mutations that alter conserved m^6A motifs in human cancer

Artz *et al.*

TABLE AND FIGURES

Tables can be found in the supplemental Excel file (Artz_m6A_supp_tables.xlsx)

Supplemental Table S1. Details on the studies used to compile the m^6A site data set.

Supplemental Table S2. List of patients excluded from the TCGA mutational data set due to their MSI-H (TCGA-MSI) or POLE (TCGA-POLE) status.

Supplemental Table S3. Compiled data base of unique m^6A sites used in this study.

Supplemental Table S4. Differentially expressed genes with gained m^6A site.

Supplemental Figure 1

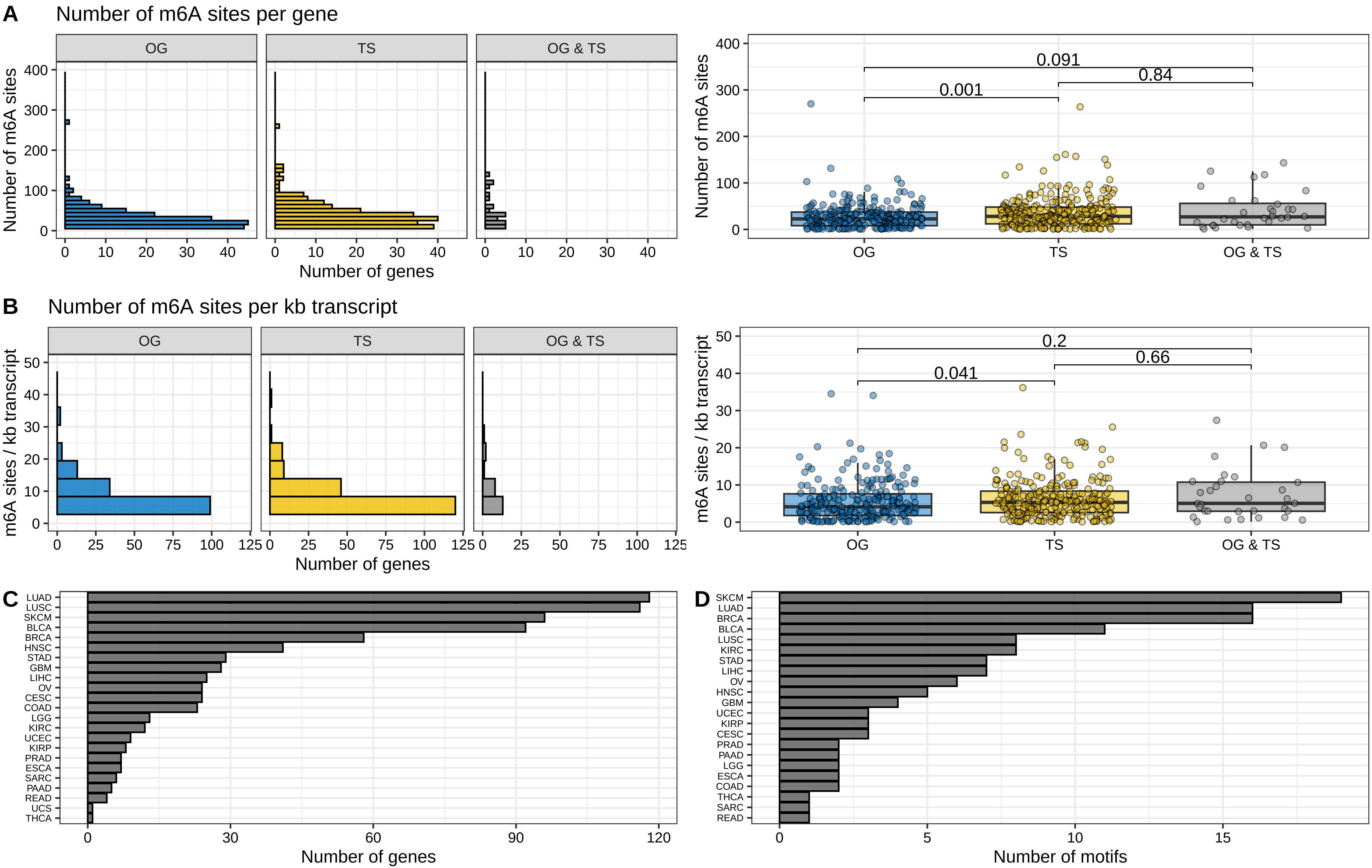

Supplemental Figure S1. Overview of number of m6A sites and m^6A site disruptions. (A + B) Left: Histogram of m^6A sites in genes classified as oncogenes (OG), tumor suppressors (TS), or both (OG & TS); Right: Boxplot of number of m^6A sites in genes classified as OG, TS, or OG & TS, p-values were determined using the Mann-Whitney U test. (A) depicts the number of m^6A sites per gene, (B) depicts the number of m^6A sites per gene, normalized by transcript length including coding sequence and untranslated regions (UTR). (C) Number of genes with mutations that disrupt m^6A sites recurring in at least two patients. (D) Number of motifs with mutations that disrupt m^6A sites recurring in at least two patients.

Supplemental Figure 2

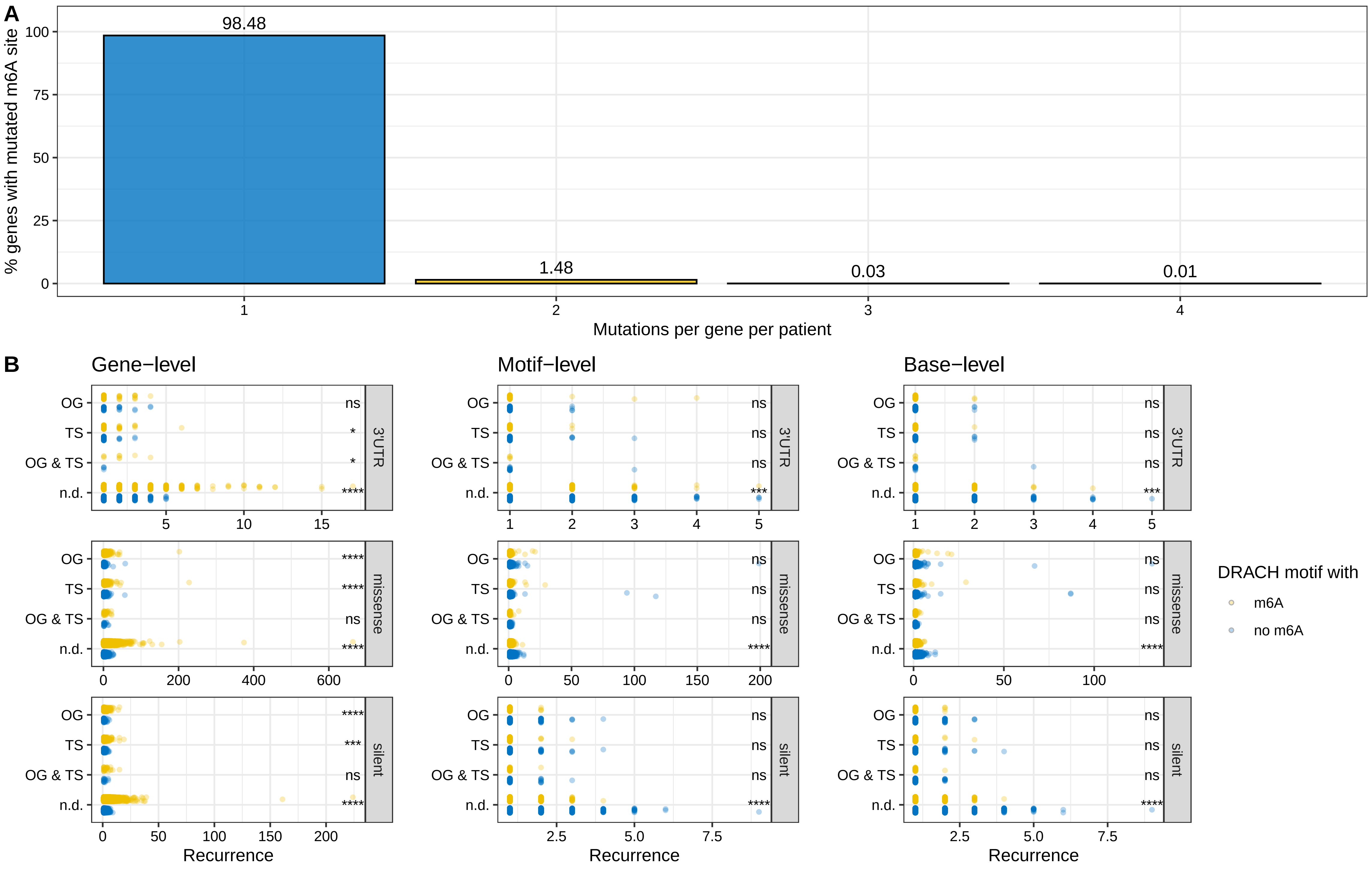

Supplemental Figure S2. Disrupted m^6A sites per patient and recurrence of DRACH motif methylation. (A) For each patient the number of mutations disrupting m^6A sites per gene was counted. The bars represent the percentage of all genes in all patients harboring the indicated number of mutations. The numbers above the bars represent the rounded percentage. (B) Genes were classified as oncogenes (OG), tumor suppressors (TS), both (OG & TS), or neither (n.d.). The recurrence of mutations disrupting m6A sites was counted for individual genes (gene-level), motifs (motif-level), and bases (base-level). Missense, 3'UTR, and silent mutations are shown separately. Statistical significance was determined using the Mann-Whitney-U test ($p<0.0001$ = ****, $p<0.001$ = ***, $p<0.01$ = **, $p<0.05$ = *, $p>=0.05$ = n.s.)

Supplemental Figure 3

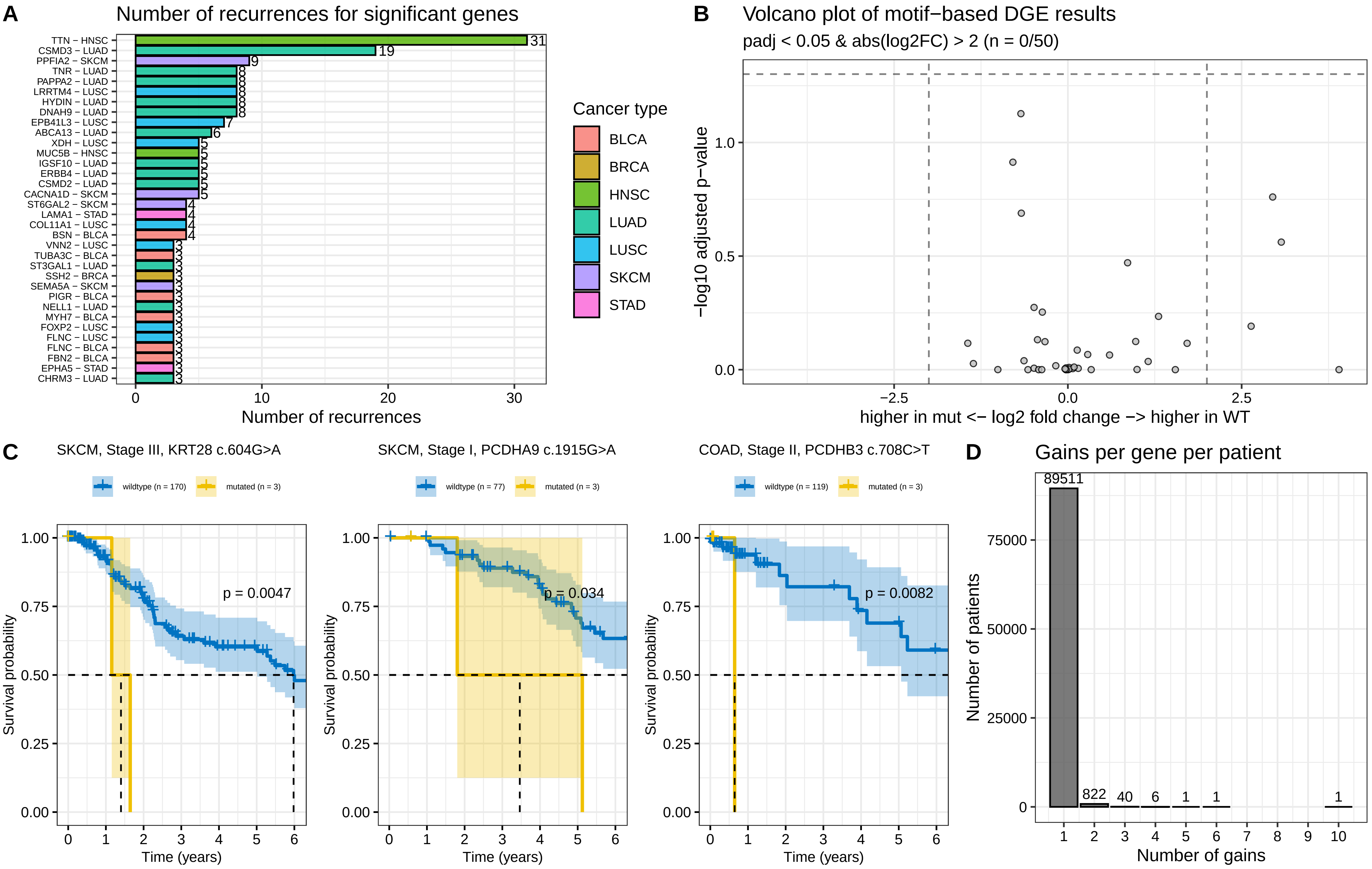

Supplemental Figure S3. Recurrence and effect of gained DRACH motifs. (A) Number of recurrent DRACH gains for genes with a significant effect in transcript abundance. Y-axis labels depict the gene name and cancer type for which a significant result was found. Numbers next to the bars represent total the total number of recurrences. (B) Motif-level analysis of transcript abundance. Genes with gained m^6A motifs in at least two patients were considered in the analysis. (C) Kaplan-Meier curves for patients with or without specific mutational gain. (D) Number of gains per gene per patient. The numbers on top of the bars indicate the number of patients that were identified for respective number of gains.
